# Mental health and wellbeing of seafaring personnel during COVID‐19: Scoping review

**DOI:** 10.1002/1348-9585.12361

**Published:** 2022-09-22

**Authors:** Samantha K. Brooks, Neil Greenberg

**Affiliations:** ^1^ Department of Psychological Medicine, King's College London Weston Education Centre London UK

**Keywords:** COVID‐19, maritime health, mental health, pandemic, seafarers, wellbeing

## Abstract

**Objectives:**

We aimed to synthesize published literature on seafarers' mental health and wellbeing during the COVID‐19 pandemic.

**Methods:**

This scoping review searched four electronic databases for literature on the mental health and wellbeing of seafarers during the COVID‐19 pandemic.

**Results:**

Fourteen studies were included in the review. Few reported on the prevalence of mental health conditions. Only one compared mental health data gathered during the pandemic to pre‐pandemic matched samples, suggesting symptoms of depression and anxiety were greater during the pandemic. There was some evidence that mental health worsened with longer stays on board during the pandemic and being on board longer than expected. Crew exchange difficulties forced many participants to extend their contracts or delay repatriation, often with little information as to when they might get to go home, leading them to feel they had no control over their lives and causing concern about fatigue and the potential for accidents and injuries. Participants described other challenges such as denial of shore leave; concerns about finances and future employment; loneliness and isolation; fears of COVID‐19 infection; limited access to essential supplies; and feeling unsupported by management.

**Conclusions:**

Maritime organizations must understand how best to support their staff in the aftermath of the COVID‐19 pandemic and in any other prolonged crises that may arise in the future. Recommendations include ensuring that staff feel valued by their organization; enhancing work‐related autonomy; ensuring that communication is accurate, consistent, and timely; and using lessons learned from the COVID‐19 pandemic to inform emergency preparedness policies.

## 1 INTRODUCTION

Personnel in seafaring professions experience various potential hazards and stressors in the workplace, including exposure to poor physical conditions, long working hours, and social isolation, all of which can affect their physical and mental health.^1^ Previous research has suggested that seafarers are at high risk for mental health problems and suicide.^2^ The most recent literature review on seafarers' mental health,^3^ which reviewed literature published between 2012–2021, identified numerous risk factors for poor mental health among seafarers, including prolonged exposure to noise and vibration; feeling unsafe onboard; high job demands; long working hours; night shifts or irregular shifts; poor sleep; poor cohesion between team members; poor management; poor social support; lack of autonomy at work; scheduling uncertainties; long duration at sea; and over‐commitment to the job.

It seems logical to assume that the COVID‐19 pandemic may have exacerbated much of the stress experienced by seafarers: research suggests that the pandemic has had a negative impact on mental health in the general population^4^ and should be understood as a potential traumatic stressor.^5^ Due to much of the world's trade depending on seafarers, they have had to continue working throughout the pandemic. While the entire world has had to endure pandemic‐related restrictions and adjust to new ways of living and working, seafarers have been in a particularly unique position; they are simultaneously more isolated than the majority of people (given that, at sea, they are ‘cut off’ from the outside world) whilst also less able to have their own space away from other people, as they are living and working alongside other crew members 24/7 in close quarters. Indeed, the crowded, enclosed nature of a ship makes it easy for COVID‐19 to spread and many ships saw outbreaks of COVID‐19 due to challenges in contact tracing, language barriers, and complex command lines negatively affecting communication about protective measures, and the crowded living conditions on board making it difficult to implement social distancing measures.^6^ Many of the earliest media reports of the pandemic focused on outbreaks on cruise ships such as the Diamond Princess, with anecdotal data suggesting that many of the crew experienced adverse effects such as exhaustion, emotional instability, and insomnia.^7^

Reports emerging from the early days of the pandemic suggested that seafarers were facing a number of challenges: first, restrictions prevented seafarers from disembarking to carry out crew changes, meaning many were ‘stranded’ aboard.^8^ In addition to crew change difficulties, seafarers were affected by lack of access to medical care; limited medical facilities and equipment on board; extended periods at sea beyond their contract length; conflicting information from different sources; pressure from family to return home; concern for family members' health; concerns about financial instability; and difficulty getting shore leave.^9^ By April 2020, the International Seafarers Welfare and Assistance Network (ISWAN) reported that the volume of calls to their SeafarerHelp helpline had tripled^10^ and in October 2020 they reported that some of the main issues their callers discussed were financial difficulties as a direct result of the pandemic, repatriation issues, mental and physical health concerns and unpaid wages.^11^ In December 2020, the International Labour Organization (ILO) ruled that governments had failed to meet their duty of care to seafarers during the pandemic as required by law, in terms of their rights to healthcare, repatriation, and shore leave.^12^ The new variants of COVID‐19 which emerged throughout 2021 led to governments imposing more travel restrictions and so the ‘crew change crisis’, while lessened, was not resolved.^13^

The most recent review of seafarers' mental health^3^ identified only two COVID‐19‐related studies, as the searches were carried out in early 2021 at which point few empirical studies on seafarers' experiences of the pandemic had been published. The results of the two studies included in the existing review suggested that the pandemic had adversely affected seafarers' mental health.

The rationale for the current scoping review is as follows: (1) the COVID‐19 pandemic has been a source of stress for many people across the world and there is evidence to suggest there are negative mental health effects of the pandemic^4^; (2) those in seafaring occupations have experienced significant stressors during the pandemic which are unique to seafaring^11^ and therefore it is important to consider seafarers as a unique population in order to understand the impact of these stressors on their wellbeing; and (3) there has, until now, not been an attempt to collate all existing research relating to seafarers' COVID‐19 experiences and therefore a review is needed.

The aim of the current scoping review was to assess how many empirical studies on seafarers' pandemic experiences have now been published and to collate their data in order to provide a clearer picture of how seafarers' mental health and wellbeing have been affected by COVID‐19.

## 2 METHODS

This review was carried out according to the guidelines outlined in Arskey and O′Malley's scoping review framework.^14^

First, the research question was identified: *What impact has the COVID‐19 pandemic had on the mental health and wellbeing of seafarers, and what factors might be associated with this impact?*

Second, to identify relevant studies, we designed the following search strategy:

(seafarer* or seafaring or sea‐farer or sea‐faring or Navy personnel or Marines or maritime or sailor* or seamen or seaman or mariner* or cruise ship staff or cruise ship worker* or cruise ship personnel or shipper* or shipping industry) AND (covid* or coronavirus or sars‐cov‐2)

This combination of terms was used to search four electronic databases (Web of Science, Embase, PsycInfo, and Medline) in April 2022; we did not limit the searches by date. All resulting citations were downloaded to EndNote reference management software where duplicates were immediately removed.

Third, we developed a set of inclusion criteria to select studies relevant for the review. To be included, studies had to: be published in the English language, as this is the language spoken by the reviewers; contain primary quantitative or qualitative data on the impact of COVID‐19 on seafarers' mental health or wellbeing; have a sample size greater than 1 (i.e. no case studies); be published in peer‐reviewed journals (i.e. no gray literature); and include data on seafarers who work at sea for prolonged periods of time (i.e. personnel who work on boats by day but go home at night would not be included). The titles of all downloaded citations were screened for relevance, with any clearly not meeting the inclusion criteria excluded from the review. Next, abstracts were screened against the inclusion criteria, and finally, full texts of all remaining citations were screened. At this stage, the reference lists of studies meeting all the inclusion criteria were hand‐searched to identify any additional relevant studies which did not appear in our database searches. All screening was carried out by the first author.

In order to carry out systematic data extraction, a spreadsheet was designed to collect the following data for each study: Authors, year of publication, country of authors, time‐point of data collection, number of participants, socio‐demographic characteristics of participants (age, gender, nationality, type of ship worked on), variables measured, tools used, and key results. The first author carefully read each included paper and extracted this information from each, completing each column in the spreadsheet. Thematic analysis^15^ was used to group the results of the included studies into themes. Finally, the results of the thematic analysis were summarized, and below we present a narrative description of the evidence found within each theme.

## 3 RESULTS

**FIGURE 1:**
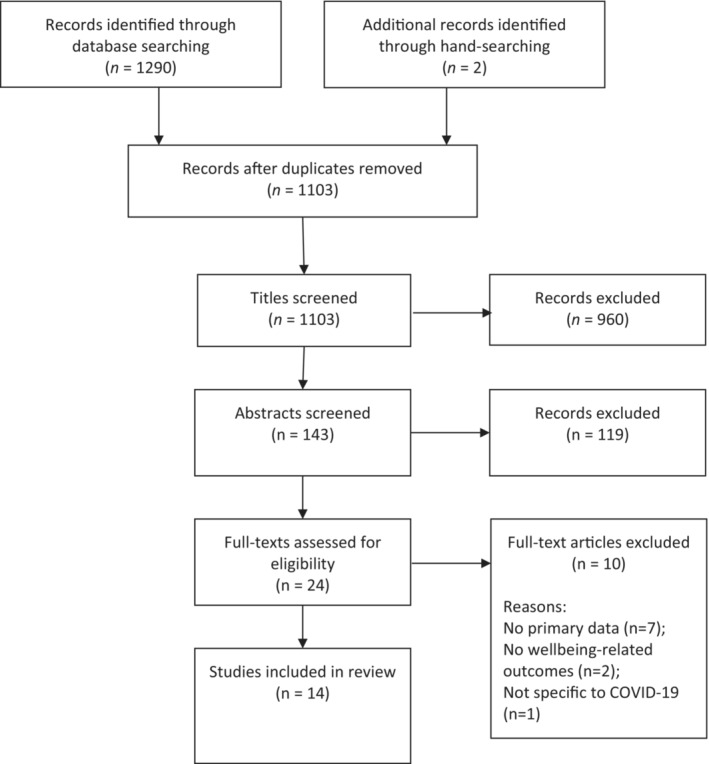
Screening process

The database searches yielded 1290 articles, of which 189 were duplicates and immediately removed. Title screening resulted in the exclusion of 960 articles, and abstract screening resulted in the exclusion of a further 119, leaving 22 full texts to be screened. Ten were excluded after reading the papers in their entirety, and two additional studies were found via hand‐searching the reference lists of included papers, resulting in 14 studies for review.^16^, ^17^, ^18^, ^19^, ^20^, ^21^, ^22^, ^23^, ^24^, ^25^, ^26^, ^27^, ^28^, ^29^ A diagram of the screening process is presented in Figure 1. An overview of the characteristics of these studies is presented in Table 1.

**TABLE 1:** Characteristics of included studies

Authors (year)	Country of authors	Participants: ‘n’ and job role	Participant demographics	Relevant measures (dates of data collection)
Battineni et al.^16^	Italy	1458 crew members of merchant ships	85.11% male, 14.89% female Mean age not reported; the largest age group represented was 40–50 (39.2%) Nationality of participants not reported	Survey assessing demographics; personal characteristics; knowledge of COVID‐19 Dates of data collection not reported
Baygi et al.^17^	Iran	439 multinational seafarers working on two oil tanker international shipping companies	Gender not reported Mean age 34.5 Majority (77.7%) were Indian	Surveys assessing psychosocial distress; general mental health; anxiety; perceived health status Data collected July 2020
Baygi et al.^18^	Iran	439 multinational seafarers working on two oil tanker international shipping companies	Gender not reported Mean age 34.5 Majority (77.7%) were Indian	Surveys assessing general anxiety disorder; post‐traumatic stress disorder; depressive symptoms Data collected July 2020
Coutroubis et al.^19^	UK & Cyprus	400 seafarers on 76 commercial vessels & 100 seafarers awaiting employment	Gender not reported Age not reported Mostly (*n* = 232) from the Philippines	Seafarers in employment: Study‐specific survey assessing when and where they joined the vessels and original duration of contract; normal vacation periods between contracts; when they expected to return home; how they reacted to extension of stay; time spent in ports and anchorages; lockdown situation in home country; family commitments; concerns about COVID‐19; concerns about the threat of COVID‐19 to family and to life on board; concerns about practical and emotional consequences of COVID‐19 Seafarers awaiting employment: Study‐specific survey assessing period of waiting between employments; whether the pandemic delayed joining vessel; family pressures due to employment delays; financial necessity of seeking alternate employment Data collected May–June 2020
Devereux & Wadsworth^20^	UK	352 British seafarers	85% male, 14% female, 1% other Mean age 36 100% British	Study‐specific questionnaire assessing health, safety, and wellbeing experiences during the pandemic Data collected June–August 2020 and January–March 2021
Hebbar & Mukesh^21^	Sweden	288 seafarers, 18 shipping companies (including 4 ship‐management companies), 6 maritime administrations	Gender not reported Age not reported Nationality not reported	Seafarers: Questionnaire assessing experiences relating to their rights to shore leave, repatriation, and medical assistance Companies and administrations: Questionnaire assessing guidelines, response action, and coordination with stakeholders Data collected June–August 2020
Lucas et al.^22^	France & Denmark	‘n’ not reported Psychological phone consultations with 32 seafarers from France; also phoned social relations of 3 seafarers, 2 retired seafarers, 2 foreign crew members, and all crew members on 3 ships	Gender not reported Age not reported Majority from France	Telephone consultations/interviews to assess the impact of COVID‐19 and onboard accidents Data collected November 2020
Pauksztat et al. (2022a)^23^	Sweden & Australia	1297 seafarers who worked on international commercial vessels	96.1% male Mean age 36.8 68.1% from Asia o Middle East; 28.1% European nationals	Surveys assessing symptoms of anxiety; symptoms of depression; how frequently symptoms of anxiety and depression had been experienced; socio‐demographic characteristics; work characteristics (experience at sea, expected length of current work period, months on board, time spent over contract, number of port calls used as a proxy for workload, ship type, vessel's flag state); severity of the impact of the COVID‐19 pandemic on work and life on board Data collected July–September 2020 and compared with matched samples of pre‐COVID data from 2015 to 2016
Pauksztat et al. (2022b)^24^	Sweden & Australia	622 seafarers on international commercial vessels	94.7% male Mean age 40.24 Participants came from 40+ countries on 6 continents, most (34.1%) from the Philippines, followed by Denmark, Germany, and Sweden (7.4% each)	Surveys assessing mental health problems (symptoms of depression and anxiety); fatigue; how frequently mental health symptoms and fatigue had been experienced in the past week; study‐specific questions assessing the impact of COVID‐19 on work and life on board and on seafarers' employment and family concerns; length of time on board; whether seafarers had been on board longer than expected; onboard peer support; level of external support; internet quality; socio‐demographics Data collected July–September 2020
Pesel et al.^25^	Italy, Spain & Denmark	72 seafarers on container ships	100% male Mean age 39 International ‐ 54% from Asian countries, 17% from European countries, 28% from Russian and former USSR countries, and 1.3% other.	GHQ‐12 to assess mental health, with three extra questions about COVID‐19 precautions on board Data collected January–April 2020
Qin et al.^26^	China	441 seafarers across 30 ships	100% male Mean age 37.54 100% Chinese	Self‐administered questionnaire assessing socio‐demographics; occupational characteristics (sailing duration, type of ship, working position, night shift frequency per week, work stress); health‐related behaviors (self‐rated health, history of chronic disease, cigarettes, alcohol, sleep duration, sleep quality, leisure time, physical exercise); depression; COVID‐19 stress Data collected June–July 2020.
Radic et al.^27^	Croatia, New Zealand, Spain, Chile & South Korea	9 cruise ship workers on 9 different cruise ships during the pandemic	44.4% male, 55.6% female Mean age 34.3 Nationalities: Philippines (3), Croatia, India, Indonesia, Serbia, Trinidad, and Tobago, UK (1 each)	Online focus group to explore the psychological effects of the COVID‐19 pandemic on cruise ship employees stuck at sea Data collected May 2020
Shan^28^	Canada	29 (7 seafarers working on board; 9 seafarers waiting to join the ship; 5 ship managers; 4 union representatives; 4 key informants from maritime welfare/maritime authorities)	Gender not reported Age not reported Participants were from Canada, China, or India	Interviews exploring the challenges faced during the pandemic with regard to 4 aspects: impact of COVID on employment, health, and safety challenges at sea, impact of public health measures on crew exchange and shore leave, and resources/support available Media coverage analysis Data collected June–December 2020
Sliskovic^29^	Croatia	752 seafarers employed in the global shipping sector	89.2% male Mean age 37.34 Participants from 57 countries	Study‐specific questionnaire with questions on sociodemographic and work characteristics and one open question about personal experiences of the pandemic Data collected April–May 2020

The following themes emerged from the data: prevalence of mental health problems, stress, and negative emotional states; potential predictors of mental health; crew exchange challenges; denial of shore leave; finances and future employment; loneliness, isolation, and missing family; fears of COVID‐19 infection; access to supplies; and perceptions of leadership and shipping companies.

### 3.1 Prevalence of mental health problems, stress, and negative emotional states

Whilst many participants described negative emotional states and being under extreme stress, only four studies^17^, ^18^, ^23^, ^26^ used standardized assessments of mental disorders such as post‐traumatic stress disorder (PTSD), depression, and anxiety. Only one study with mental ill‐health prevalence data compared data collected during the pandemic with data collected several years earlier, finding higher levels of symptoms during the pandemic than before.^23^

#### 3.1.1 Post‐traumatic stress symptoms

Baygi et al.^18^ found that 37% of their 439 participants had disruptions within at least one of the three PTSD domains (intrusion, hypervigilance, and avoidance), with approximately 12% experiencing disruption within all three. Whilst Lucas et al.^22^ had no quantitative measure of PTSD, they reported that many seafarers who called their health recourse center described symptoms of severe PTSD.

#### 3.1.2 Anxiety

Approximately 11–12% of Baygi et al.’s^17^, ^18^ 439 participants reported symptoms of anxiety, although only 2% self‐identified as ‘anxious'.^17^ Pauksztat et al.^23^ found that seafarers surveyed during the pandemic had significantly higher levels of anxiety than a matched sample who responded to surveys during 2015–2016.

#### 3.1.3 Depression

Rates of depressive symptoms ranged from 12–14% (of *n* = 439)^17^, ^18^ to approximately 42% (of *n* = 441);^26^ in the latter study, 9% reported severe depressive symptoms. All nine of the participants in Radic et al.’s study^27^ reported at least some depressive symptoms, although there was no quantitative measure of depression in this study. Pauksztat et al.^23^ found that seafarers surveyed during the pandemic had significantly higher levels of depression than a matched sample who responded to surveys during 2015–2016.

#### 3.1.4 General mental health

Over 40% of Baygi et al.’s^17^ participants reported general psychiatric disorders. Pauksztat et al.^24^ found that on average respondents had experienced symptoms of mental health problems and fatigue ‘once’ or ‘several times' in the past week, although there was considerable variation between participants. One study^16^ found that almost 60% (of *n* = 1458) of participants reported feeling ‘mentally well’.

#### 3.1.5 Stress

Prevalence of stress varied. Baygi et al.^17^, ^18^ found that stress was reported by 6% (of *n* = 439) participants; Battineni et al.^16^ found that 11.5% (of *n* = 1458) reported feeling ‘over‐stressed’; and Hebbar and Mukesh^21^ found that approximately 30% (of *n* = 288) felt stressed. Eight of the nine participants in Radic et al.’s^27^ qualitative study reported feeling ‘stressed or nervous'. Meanwhile, 85% of Coutroubis et al.’s^19^ 400 participants reported being concerned about fellow crew members' mental stress.

#### 3.1.6 Sleep and fatigue

Approximately one‐sixth of Hebbar and Mukesh's^21^ 288 seafarers reported feeling ‘completely fatigued’, while all nine of Radic et al.’s^27^ participants reported their sleep was poor due to worries, fears, and anxieties, and 30% of Pesel et al.’s^25^ 72 participants suffered insomnia to the extent of becoming concerned. Sleep disturbances, fatigue, and exhaustion were also reported by participants in three other studies, although no statistics were provided.^22^, ^28^, ^29^ Participants expressed concerns that fatigue had adversely affected their physical and mental health^29^ and that accumulated fatigue could lead to poor concentration, poor judgment, and potential accidents or injuries.^28^

#### 3.1.7 (Un)happiness

Approximately 40% of Hebbar and Mukesh's^21^ 288 seafarers reported feeling unhappy; only 8% reported feeling happy regardless of circumstances. 26% of Pesel et al.’s^25^ 72 participants reported they had been unhappy and depressed during their latest tours of duty; almost half felt less happy than usual, and 40% felt less able to find and enjoy free time than pre‐pandemic.

#### 3.1.8 Other

Some participants (no statistics provided) described other negative emotional states such as worry, mental tiredness, nervousness, and sadness;^29^ feeling they had no control over their lives;^27^ and feeling that their psychological capacities for adaptation had been overloaded.^22^ Many felt their mental and physical state negatively affected their motivation to work, leaving them unable to concentrate or engage in work tasks.^29^

### 3.2 Potential predictors of mental health

#### 3.2.1 Marital status

Baygi et al.^18^ found that depressive symptoms and hypervigilance symptoms were significantly higher among married personnel, although married personnel had significantly better perceptions of their health status.^17^ Marital status was not associated with the severity of depression in Qin et al.’s study.^26^

#### 3.2.2 Age

Baygi et al.^17^ found that anxious crew members were significantly older than non‐anxious. However, age was not associated with the severity of depression in Qin et al.’s study.^26^

#### 3.2.3 Other socio‐demographic characteristics

Educational level and income were not associated with the severity of depression in Qin et al.’s study.^26^

#### 3.2.4 Health and lifestyle

Qin et al.^26^ found that poorer self‐rated health, engaging in less leisure activities or physical exercise per week, poor sleep quality, sleep duration of less than 6 h, and high self‐perceived work stress were associated with greater severity of depressive symptoms. In the same study, chronic disease, smoking cigarettes, drinking alcohol, and COVID‐19‐related stress were not associated with the severity of depression.

#### 3.2.5 Rank

Baygi et al.^18^ found that depressive symptoms and all three PTSD sub‐scales were significantly higher among officers than non‐officers, whilst Baygi et al.’s earlier study^17^ found general psychiatric disorders, anxiety, and stress were significantly greater among officers. Pauksztat et al.^23^ found that personnel at higher hierarchical levels reported a higher impact of the pandemic. However, being a senior officer or non‐senior crew member was not associated with the severity of depression in Qin et al.’s study.^26^

#### 3.2.6 Workload

Hypervigilance and avoidance were significantly higher among those with less mean work per week,^18^ although in Qin et al.’s study,^26^ the more frequent the overtime work, the higher the level of depression.

#### 3.2.7 Type of ship

Those working on passenger vessels and on vessels with Flags of Convenience (that is, ships registered in a country other than that of the ship's owners) reported a higher impact of the pandemic,^23^ and those working on vessels registered with Flags of Convenience reported significantly higher levels of both depression and anxiety during the pandemic, but not prior to the pandemic.^23^

#### 3.2.8 Impact of the pandemic

More severe impact of COVID‐19 on ‘employment and family concerns’ and ‘work and life on board’ was associated with mental health problems and fatigue.^24^

#### 3.2.9 Support

Pauksztat et al.^24^ found that poor perceived onboard support and poor perceived external support were associated with mental health problems and fatigue.

#### 3.2.10 Time spent on vessels

Longer stay duration on board during the pandemic was significantly associated with greater intrusion symptoms,^18^ depression,^17^ mental health problems, and fatigue.^24^ Multivariate analysis by Baygi et al.^17^ found that the odds of experiencing depression increased by 20% per month staying on board. Pauksztat et al.^23^ found that seafarers with longer work periods and who had been on board longer than expected reported significantly more severe pandemic impact and significantly higher levels of both depression and anxiety during the pandemic, whereas the length of work periods and time on board were not associated with depression or anxiety pre‐pandemic. However, in one study^26^ sailing duration was not associated with the severity of depression.

### 3.3 Crew exchange challenges

The pandemic made crew exchanges both more difficult and more expensive.^28^ Crew exchange problems had led to many participants in many studies experiencing extensions to contracts and repatriation delays which they perceived to have adversely affected their mental health and wellbeing.^20^, ^21^, ^23^, ^28^, ^29^ Tours of duty were often extended over and above the extensions permitted by their contracts, against the seafarers' free will, and without compensation;^20^, ^21^ many continued working even after contracts expired as they did not want to let their companies down and also feared being blacklisted and struggling to find jobs in future if they did not comply.^28^ Others were unable to join vessels and so were not getting paid; participants with single‐voyage contracts were less likely to have joined vessels as normal and more likely to have not joined their vessel as scheduled and not been paid.^20^

There were numerous challenges relating to crew exchange issues and subsequently extended tours; uncertainty appeared to be a particular challenge, with participants receiving contradictory or incomplete information about landing possibilities^22^ and not knowing when their onboard work period would end,^29^ both of which were perceived to adversely affect mental health. Participants also reported concern that extended contracts would lead to exhausted crew which could in turn lead to accidents.^28^

Several of the participants in Devereux and Wadsworth's study^20^ reported feeling that crew changes were possible but that shipping companies were using the pandemic as an ‘excuse’ to reduce crew changes and require longer tours of duty, and reported a perceived lack of control regarding when crew changes would happen.

### 3.4 Denial of shore leave

Participants also described shore leave being canceled or denied.^21^, ^28^ Approximately three‐quarters of Hebbar and Mukesh's^21^ 288 seafarers accepted the restrictions and did not wish to have shore leave, as they feared becoming infected with COVID‐19. However, approximately five‐sixths felt their performance was affected by the absence of shore leave and three‐quarters felt the absence of shore leave had affected their health. It is also important to note that in the same study, while approximately 70% of seafarers had full awareness of the international regulations pertaining to shore leave, repatriation, and medical assistance, around 5% of participants were not aware at all of their rights.

### 3.5 Finances and future employment

Participants described concerns about their financial situation, their future employment, and the future of the industry as a whole due to the pandemic.^19^, ^20^, ^27^, ^29^ 84% of Coutroubis et al.’s^19^ 400 seafarers had concerns about future employment, whilst 90% were concerned about the global economy and trade. Many of Radic et al.’s^27^ participants described uncertainty about their future financial situation and ability to support their families, with this uncertainty leading to feelings of agitation and despair. However, some participants also reported feeling satisfied and privileged to be working and earning whilst others around the world had lost their jobs.^29^

Devereux and Wadsworth^20^ found that participants with single‐voyage contracts were significantly more likely to report the pandemic had negatively affected their career and their finances than those with permanent contracts and were also more likely to report being likely to leave the industry as a result of their pandemic experiences. Additionally, two studies^19^, ^29^ included among their participants samples of seafarers who were not on board but waiting to start their next employment. Over half of Coutroubis et al.’s^19^ 100 seafarers waiting to start their next employment reported their employment had been delayed due to the pandemic, and 82% felt concerned about delays to employment; only 4% stated they were not interested in seeking alternative options whilst waiting for their next employment, suggesting financial pressure was high. Most of Sliskovic's^29^ participants awaiting their next tour expressed concerns about economic wellbeing, reporting fears for their financial security as they were paid only during work periods and felt helpless as they did not know when they would next be paid; those in the passenger‐shipping sector also expressed fears about whether the cruise industry would ever recover.

### 3.6 Loneliness, isolation, and missing family

The prevalence of loneliness varied across studies. Only 2% of Battineni et al.’s^16^ 1458 crew members reported feeling lonely, whilst 27% reported missing family or friends; however, 91% of Coutroubis et al.’s^19^ 400 seafarers reported missing family more than usual and 80% felt they were more isolated than the rest of the world. In the latter study, the majority expressed some level of concern about a family member falling ill while they were away or family members' mental stress. Sliskovic's^29^ participants reported that the pandemic had exacerbated feelings of loneliness and missing families. Many of Radic et al.’s^27^ participants described missing family and friends, which was made worse by inability to communicate with them due to poor internet; around half of Coutroubis et al.’s^19^ 400 participants also reported finding it difficult to communicate with home, although half reported no such problems. Several of Radic et al.’s^27^ participants also described feeling lonely and depressed due to losing their usual opportunities for socializing such as the gyms and bars usually available to crew.

Some participants also described a sense of societal isolation: Lucas et al.’s^22^ participants reported both physical and societal isolation, while Sliskovic's^29^ participants described a sense of prolonged isolation from the community and abandonment by formal organizations in charge of caring for seafarers, leading to them feeling ‘forgotten’.

Additionally, feelings of isolation in Shan's^28^ study were particularly prevalent for Chinese seafarers, who described experiences of discrimination, stigma, and racism, and felt they were treated as suspected cases of COVID‐19 on board; Chinese seafarers also reportedly faced tougher restrictions and port inspections.

### 3.7 Fears of COVID‐19 infection

Shan^28^ identified ‘infection risk’ as one of the key problems facing seafarers, who reported various challenges relating to prevention measures including shortage of personal protective equipment (PPE) on board, visits from shore‐based personnel who refused to wear PPE, and companies not allowing them to wear masks (for example, one participant who worked on a cruise ship reported that masks were forbidden as they were perceived as affecting the ‘smile service’ of the crew). 95% of Coutroubis et al.’s^19^ 400 participants were worried they or another crew member would catch COVID‐19 at sea; in particular, they were worried about shoreside staff bringing the virus on board when they visited or on‐signers bringing the virus aboard when ports opened. 86% also felt concerned about infection when traveling home at the end of their contracts or extensions. In Pesel et al.’s^25^ study, half (of *n* = 72) reported not feeling safe doing their job in relation to the pandemic, and 60% felt not everything had been done to sufficiently protect their health at work. However, most of Hebbar and Mukesh's^21^ participants felt their vessels were adequately equipped to deal with COVID‐19, and approximately half trusted the sufficiency of their policies for dealing with infections. Whilst many of Sliskovic's^29^ participants reported fearing COVID‐19 infection, these fears appeared to depend on protective measures on board, with some reporting they felt safer on board than they would at home.

### 3.8 Access to supplies

Almost half of Coutroubis et al.’s^19^ 400 seafarers reported difficulty with food provision, and many of Shan's^28^ participants reported that port restrictions limited their access to healthy food such as fresh vegetables. 15% of Hebbar and Mukesh's^21^ 288 participants felt they were not provided with medical assistance ashore, and many of Shan's^28^ participants reported that port restrictions limited their access to medical care, and described how requests to take shore leave in order to see doctors, get flu shots or refill prescriptions had been denied.

### 3.9 Perceptions of leadership and shipping companies

37% of Hebbar and Mukesh's^21^ 288 seafaring participants felt the relief and repatriation efforts by their company were either non‐existent or insufficient, or that the company appeared to be helpless. However, those working in management or administration for shipping companies suggested that whilst it was a challenge to send crew home, most owners, and charterers were supportive regarding diverting vessels for crew change and repatriation.

Devereux and Wadsworth's^20^ participants reported that terms and conditions relating to additional work had been amended, meaning seafarers did not accrue leave for additional days worked on board, leading to feelings that their companies did not care about them; some reported being threatened with termination of their employment if they did not agree to extended contract terms. Participants in the same study reported that the use of temporary contracts allowed companies to no longer pay seafarers at the end of their contract even when they remained on board, and that some companies terminated the employment of permanent employees and replaced them with temporary staff on lower pay.

Many of Sliskovic's^29^ participants felt unsupported by management and highlighted the contrast between being classed as key workers and feeling like prisoners on board with no idea when they would be able to go home. 8/9 of Radic et al.’s^27^ participants were skeptical about the leadership of cruise companies and did not feel they were doing everything in their power to get seafarers home; they suggested cruise line companies had poor human resource management strategies and no contingency plans for a crisis such as the pandemic. Some reported this led them to lose faith in their organizations; however, others reported they felt looked after on board and most participants still felt willing to defend the image of cruise line companies from negative media publicity.

## 4 DISCUSSION

This review is the first to synthesize findings from all of the literature published so far on the impact of the COVID‐19 pandemic on seafarers' mental health and wellbeing. The results identified various challenges faced by seafarers during this period, although there is not yet enough literature to provide an accurate estimate for the prevalence of mental ill‐health in seafarers during the pandemic. Only four of the reviewed studies included standardized measures of mental health problems, and results varied fairly widely across these studies. Overall, the rates of anxiety and depression reported in the included studies appeared to be lower than estimates of anxiety and depression in the general population during the early months of the pandemic^30^ and did not appear particularly different from pre‐pandemic rates of anxiety and depression in seafarers;^31^ however, the only study included in this review which compared mental health during the pandemic to a matched sample from pre‐COVID‐19^23^ found that seafarers' mental health was significantly worse in 2020 than in 2015–2016. Also, the only study which assessed the prevalence of PTSD symptoms^18^ revealed that 37% experienced clinically relevant symptoms of at least one of the PTSD domains; this is higher than estimates of PTSD in seafarers after maritime piracy.^32^ Further research on the prevalence of mental ill‐health in seafarers during the pandemic is needed, in particular research that compares rates of mental health symptoms to pre‐COVID‐19 rates.

The studies included in this review provided relatively inconsistent results regarding the predictors of mental health in seafarers. There was some evidence that officers fared worse than non‐officers, and evidence that longer time on board during the pandemic (and especially longer time than originally expected) contributed to poor mental health. The latter finding is unsurprising given that mandatory tour extensions have been reported to be a cause of poor wellbeing in seafarers even in ‘normal’, pre‐pandemic times.^33^ Having tour lengths altered has also been noted as a risk factor for poor mental health in other professions who go on operational deployments, such as diplomats^34^ and military personnel.^35^

Participants described challenges with crew exchange meaning that tours of duty were extended, and many seafarers were unable to leave their ships, whilst others waiting to embark were unable to join. Those on board reported feeling compelled to extend contracts to avoid letting down their companies or adversely affecting their careers, but tour extensions led to uncertainty about when they would be able to go home and exhaustion due to working for longer than they had expected to. Due to this exhaustion, seafarers reported fearing accidents and injuries; indeed, sleep deprivation has been shown to negatively affect judgment and decision‐making^36^ and so extreme tiredness in the already hazardous environment of a vessel at sea could have potentially catastrophic consequences.

Several other challenges were identified in this review as negatively affecting the wellbeing of seafarers. Firstly, denial of shore leave seemingly helped to allay fears of COVID‐19 infection but also negatively affected health and work performance; this is unsurprising as it is widely recognized that shore leave is crucial for the mental health and wellbeing of seafarers.^2^, ^37^, ^38^ Secondly, seafarers expressed concerns about their finances and future employment—fears which have been echoed across the world by many occupational groups during the pandemic^39^ and which appear to be associated with poorer mental health.^40^, ^41^ Seafarers also reported loneliness and isolation – experiences that are often reported by seafarers even during ‘normal’ times,^42^ but which seemed to be exacerbated by the perception of being completely isolated from the ‘outside world’ during a time of crisis and the inability to socialize with crew as they normally would. Fears of COVID‐19 infection unsurprisingly appeared to be dependent on the safety measures on board and such fears appeared to negatively affect seafarers' wellbeing; fears of infection are common during pandemics and are associated with poor mental health.^40^ Lack of access to food and medical supplies was also cited as a stressor, which again has been associated with poor mental health in the general population during pandemics.^40^ Finally, many seafarers appeared to feel let down, abandoned, and unsupported by their management and their shipping companies, who were perceived as being ill‐prepared for such a crisis, although some still expressed loyalty and reported being prepared to defend their organizations from negative claims.

The results of this review have a number of implications for managers of seafaring organizations in terms of supporting staff in the aftermath of the COVID‐19 pandemic and preparing to support their staff in the case of future crises. Whilst a prolonged crisis such as a pandemic presents certain challenges which cannot easily be addressed—for example, managers cannot change the fact that government restrictions limit employees' opportunities for socializing and shore leave, or the fact that a virus such as COVID‐19 is a very real threat that some staff will be very fearful of—there are many steps they can take to protect the wellbeing of their staff. First, ensuring that staff knows they are valued can have an enormously positive effect on staff morale and wellbeing, and may begin to compensate for some of the pressures and uncertainties staff are likely to feel during a crisis. When employees feel appreciated, they experience greater personal outcomes such as job satisfaction and happiness as well as greater work‐related outcomes such as commitment and performance.^43^ Ways of assuring staff they are valued might include the provision of positive feedback, rewarding good performance, giving thanks to employees for involvement in difficult tasks or working in difficult circumstances, or holding morale‐building meetings with team members to celebrate achievements.^44^, ^45^

Second, increasing seafarers' sense of control and autonomy could help to compensate for the many uncertainties they are likely to experience during a prolonged crisis. Uncertainty is already recognized as a stressor for seafarers even in ‘normal times’, with seafarers reporting anxiety around last‐minute decisions, work scheduling uncertainties, and being asked to join vessels at short notice.^33^ A pandemic is by nature a time of uncertainty, and participants in the reviewed studies described uncertainties around tour lengths, when they would see their families again, whether they would get paid and the future of their own employment and the industry as a whole. As a result of these work‐related uncertainties, seafarers frequently reported feeling a lack of control and autonomy over their lives. Poor control and autonomy at work have been associated with poor mental health in many occupational groups, including seafarers,^3^ healthcare professionals,^46^ and the military.^47^ Seafaring organizations might therefore benefit from implementing strategies for enhancing autonomy even in times of crisis, such as including employees on organizational committees; having specific workgroups to promote employee involvement in decision‐making; enhancing competence in decision‐making through training and education; and allowing employees to collaboratively create plans to improve their work environment.^48^, ^49^ If seafaring organizations encouraged acceptance of the fact that some aspects of the job (ad the pandemic) are outside of employees' control, while also allowing staff to be involved with decision‐making processes where possible, this could potentially lessen the stress of those aspects of the job that cannot be controlled.

Seafarers also reported uncertainties arising from poor or inconsistent communication from their organizations. This is perhaps unsurprising as the pandemic and associated social restrictions are unprecedented and organizations are unlikely to have been prepared for a public health emergency of such magnitude. Indeed, research suggests other organizations, such as healthcare organizations, have also been slow to communicate about policies and risks.^50^ We suggest that, as far as possible, managers should ensure that communication is accurate, timely, and consistent, to avoid adding to the confusion and uncertainty felt by seafarers. We also suggest that seafaring organizations ensure that lessons are learned from the COVID‐19 pandemic; organizations should review what was done well and what was done less well in terms of implementing new policies and procedures relating to the pandemic, and put strategies in place for coping with future public health emergencies. This will benefit their seafaring staff if another health crisis were to occur; additionally, making their reviews of how COVID‐19 was managed public to employees would help to reassure them that their organizations are learning lessons from previous experiences.

### 4.1 Limitations

Although over 2 years have passed since the onset of the COVID‐19 pandemic, no longitudinal studies of seafarers' mental health during the pandemic were found, and all studies included in this review collected data very early on in the pandemic (nearly all collected data in spring/summer 2020, and only one included any data from 2021). For this reason, it is difficult to establish how the impact of the pandemic on seafarers may have changed as time went on. It may be useful to update this review in the future when more studies with longitudinal data and/or data collected later on in the pandemic have been published, in order to better understand what some of the long‐term impacts of the pandemic might be.

There are a number of limitations of the review process itself: firstly, the decision to limit the search to English‐language papers may have resulted in important studies being missed. Future reviews may consider including studies published in other languages. Relevant studies could also have potentially been missed due to the search terms used or the databases searched; future reviews could consider using broader search strategies and searching a greater number of databases, including gray literature searches. Finally, screening and data extraction was all carried out by one author; ideally, a second author would also screen and extract data from a sample of papers to ensure the reliability of the results.

## 5 CONCLUSION

This review contributes to the growing body of knowledge about the impact of the COVID‐19 pandemic on the wellbeing of seafarers by collating, for the first time, published literature on how the pandemic has affected seafarers' mental health and wellbeing. There are, so far, only a small number of studies assessing the prevalence of mental ill‐health in this population during the pandemic and only one comparing it to pre‐pandemic levels. However, studies illustrate some of the main stressors faced by this group, such as crew exchange challenges; denial of shore leave; concerns about finances and future employment; loneliness; fears of COVID‐19; lack of access to food and healthcare; and feeling unsupported by management. Many of these stressors are prevalent in seafaring populations anyway and have only been exacerbated by the pandemic. There are steps that managers of seafaring organizations can take to support their staff at this time and in future crises, including ensuring that staff feel appreciated; allowing more autonomy at work where possible; ensuring that communication is as accurate, consistent, and timely as it can be; and using lessons learned from the COVID‐19 pandemic to inform policies for dealing with emergencies and public health crises in the future.

## AUTHOR CONTRIBUTIONS

S.K.B. and N.G. conceived the idea; S.K.B. carried out data searching, screening, extraction, and analysis; S.K.B. led the writing and N.G. edited the final manuscript.

## CONFLICT OF INTEREST

Authors declare no conflict of Interests for this article.

## APPROVAL OF THE RESEARCH PROTOCOL

N/A

## INFORMED CONSENT

N/A

## REGISTRY AND REGISTRATION NO. OF THE STUDY/TRIAL

N/A

## ANIMAL STUDIES

N/A.

## Data Availability

Data sharing is not applicable to this article as no new data were created or analyzed in this study.

## References

[1] Oldenburg M , Baur X , Schlaich C . Occupational risks and challenges of seafaring. J Occup Health. 2010;52(5):249‐256. 20661002 10.1539/joh.k10004

[2] Iversen RTB . The mental health of seafarers. Int Marit Health. 2012;63(2):78‐89. 22972547

[3] Brooks SK , Greenberg N . Mental health and psychological wellbeing of maritime personnel: a systematic review. BMC Psychol. 2022 [in press];10:139. 35637491 10.1186/s40359-022-00850-4 PMC9150387

[4] Ranaei V , Pilevar Z , Aghamolaei T . Behavioral reactions and psychological responses to 2019‐nCoV: a narrative review. Iran J Psychiatry Behav Sci. 2020;14(3):7.

[5] Bridgland VME , Moeck EK , Green DM , et al. Why the COVID‐19 pandemic is a traumatic stressor. PLoS ONE. 2021;16(1):e0240146. 33428630 10.1371/journal.pone.0240146 PMC7799777

[6] Kordsmeyer AC , Mojtahedzadeh N , Heidrich J , et al. Systematic review on outbreaks of SARS‐CoV‐2 on cruise, navy and cargo ships. Int J Environ Res Public Health. 2021;18(10):26. 10.3390/ijerph18105195 PMC8153346 34068311

[7] Takahashi S , Manaka K , Hori T , Arai T , Tachikawa H . An experience of the Ibaraki disaster psychiatric assistance team on the diamond princess cruise ship: mental health issues induced by COVID‐19. Disaster Med Public Health Prep. 2020;14(4):e46‐e47. 10.1017/dmp.2020.305 PMC7477399 32782043

[8] Doumbia‐Henry C . Shipping and COVID‐19: protecting seafarers as frontline workers. WMU J Marit Aff. 2020;19:279‐293.

[9] Stannard S . COVID‐19 in the maritime setting: the challenges, regulations and the international response. Int Marit Health. 2020;71(2):85‐90. 32604450 10.5603/IMH.2020.0016

[10] ISWAN . Helpline cases triple as seafarers seek help during COVID‐19 pandemic. https://www.seafarerswelfare.org/news/2020/helpline‐cases‐triple‐as‐seafarers‐seek‐help‐during‐covid‐19‐pandemic Published April 2020. Accessed May 26, 2002.

[11] ISWAN . World mental health day – mental health for all seafarers. https://www.seafarerswelfare.org/news/2020/world‐mental‐health‐day‐mental‐health‐for‐all‐seafarers Published October 2020. Accessed May 26, 2022.

[12] ILO . General observation on matters arising from the application of the Maritime Labour Convention, 2006, as amended (MLC, 2006) during the COVID‐19 pandemic. https://www.ilo.org/wcmsp5/groups/public/‐‐‐ed_norm/‐‐‐normes/documents/publication/wcms_764384.pdf Published 2020. Accessed May 26, 2022.

[13] ISWAN . Concerns of rise in number of seafarers impacted by crew change due to new COVID‐variants. https://www.seafarerswelfare.org/news/2021/concerns‐of‐rise‐in‐number‐of‐seafarers‐impacted‐by‐crew‐change‐due‐to‐new‐covid‐variants Published March 2021. Accessed May 26, 2002.

[14] Arksey H , O'Malley L . Scoping studies: towards a methodological framework. Int J Soc Res Methodol. 2005;8(1):19‐32.

[15] Braun V , Clarke V . Using thematic analysis in psychology. Qual Res Psychol. 2006;3(2):77‐101.

[16] Battineni G , Sagaro GG , Chintalapudi N , Di Canio M , Amenta F . Assessment of awareness and knowledge on novel coronavirus (COVID‐19) pandemic among seafarers. Healthcare. 2021;9(2):25. 10.3390/healthcare9020120 PMC7912131 33503921

[17] Baygi F , Khonsari NM , Agoushi A , Gelsefid SH , Gorabi AM , Qorbani M . Prevalence and associated factors of psychosocial distress among seafarers during COVID‐19 pandemic. BMC Psychiatry. 2021;21(1):9. 33931081 10.1186/s12888-021-03197-z PMC8085649

[18] Baygi F , Blome C , Smith A , et al. Post‐traumatic stress disorder and mental health assessment of seafarers working on ocean‐going vessels during the COVID‐19 pandemic. BMC Public Health. 2022;22(1):242. 35123421 10.1186/s12889-022-12673-4 PMC8817943

[19] Coutroubis AD , Menelaou AA , Adami E‐H . Impact of coronavirus disease (COVID‐19) on Seafarers' life and well‐being. Int J Trop Dis Health. 2020;41(21):16‐27.

[20] Devereux H , Wadsworth E . Forgotten keyworkers: the experiences of British seafarers during the COVID‐19 pandemic. Econ Labour Relat Rev. 2022;33:272‐289. doi:10.1177/10353046221079136

[21] Hebbar AA , Mukesh N . COVID‐19 and seafarers' rights to shore leave, repatriation and medical assistance: a pilot study. Int Marit Health. 2020;71(4):217‐228. 33394486 10.5603/IMH.2020.0040

[22] Lucas D , Jego C , Jensen OC , et al. Seafarers' mental health in the COVID‐19 era: lost at sea? Int Marit Health. 2021;72(2):138‐141. 34212354 10.5603/IMH.2021.0023

[23] Pauksztat B , Andrei DM , Grech MR . Effects of the COVID‐19 pandemic on the mental health of seafarers: a comparison using matched samples. Saf Sci. 2022;146:105542. 34744311 10.1016/j.ssci.2021.105542 PMC8556536

[24] Pauksztat B , Grech MR , Kitada M . The impact of the COVID‐19 pandemic on seafarers' mental health and chronic fatigue: beneficial effects of onboard peer support, external support and internet access. Mar pol. 2022;137:104942. 10.1016/j.marpol.2021.104942 PMC8732879 35013636

[25] Pesel G , Canals ML , Sandrin M , Jensen O . Wellbeing of a selection of seafarers in eastern Adriatic Sea during the COVID‐19 pandemic 2020. Int Marit Health. 2020;71(3):184‐190. 33001430 10.5603/IMH.2020.0033

[26] Qin WZ , Li L , Zhu DS , Ju CF , Bi PF , Li SX . Prevalence and risk factors of depression symptoms among Chinese seafarers during the COVID‐19 pandemic: a cross‐sectional study. BMJ Open. 2021;11(6):8. 10.1136/bmjopen-2021-048660 PMC8230921 34162652

[27] Radic A , Luck M , Ariza‐Montes A , Han H . Fear and trembling of cruise ship employees: psychological effects of the COVID‐19 pandemic. Int J Environ Res Public Health. 2020;17:6741. 10.3390/ijerph17186741 PMC7559441 32947859

[28] Shan D . Occupational health and safety challenges for maritime key workers in the global COVID‐19 pandemic. Int Labour Rev. 2021;22:22. 10.1111/ilr.12220 PMC8444828 34548682

[29] Sliskovic A . Seafarers' well‐being in the context of the COVID‐19 pandemic: a qualitative study. Work. 2020;67(4):799‐809. 33325430 10.3233/WOR-203333

[30] Salari N , Hosseinian‐Far A , Jalali R , et al. Khaledi‐Paveh. Prevalence of stress, anxiety, depression among the general population during the COVID‐19 pandemic: a systematic review and meta‐analysis. Glob Health. 2020;16:57. 10.1186/s12992-020-00589-w PMC7338126 32631403

[31] Lefkowitz RY , Null DB , Slade MD , Redlich CA . Injury, illness, and mental health risks in United States domestic mariners. J Occup Environ Med. 2020;62(10):839‐841. 32769797 10.1097/JOM.0000000000001968

[32] Seyle DC , Fernandez KG , Dimitrevich A , Bahri C . The long‐term impact of maritime piracy on seafarers' behavioral health and work decisions. Mar pol. 2018;87:23‐28.

[33] Devereux H , Wadsworth E . Work scheduling and work location control in precarious and ‘permanent’ employment. Econ Labour Relat Rev. 2021;32(2):230‐246.

[34] Dunn R , Williams R , Kemp V , Patel D , Greenberg N . Systematic review: deployment length and the mental health of diplomats. Occup Med. 2015;65(1):32‐38. 10.1093/occmed/kqu142 25326909

[35] Buckman JEJ , Sundin J , Greene T , et al. The impact of deployment length on the health and well‐being of military personnel: a systematic review of the literature. Occup Environ Med. 2011;68:69‐76. 20884791 10.1136/oem.2009.054692

[36] Violanti JM , Charles LE , McCanlies E , et al. Police stressors and health: a state‐of‐the‐art review. Policing. 2017;40(4):642‐656. 30846905 10.1108/PIJPSM-06-2016-0097 PMC6400077

[37] An J , Gao W , Liu R , Liu Z . Empirical study on the relationship between vacation schedule and seafarers' fatigue in Chinese seafarer population. Frontiers Psychol. 2022;13:838811. doi:10.3389/fpsyg.2022.838811 PMC8977519 35386897

[38] Borovnik M . Occupational health and safety of merchant seafarers from Kiribati and Tuvalu. Asia Pac Viewp. 2011;52(3):333‐346. 22216477 10.1111/j.1467-8373.2011.01459.x

[39] Cech EA , Hiltner S . Unsettled employment, reshuffled priorities? Career prioritization among college‐educated workers facing employment instability during COVID‐19. Socius. 2022;8:237802312110686. doi:10.1177/23780231211068660 PMC10978048 38550749

[40] Brooks SK , Webster R , Smith L , et al. The psychological impact of quarantine and how to reduce it: rapid review of the evidence. Lancet. 2020;395(10227):912‐920. 32112714 10.1016/S0140-6736(20)30460-8 PMC7158942

[41] Wilson JM , Lee J , Fitzgerald HN , Oosterhoff B , Sevi B , Shook NJ . Job insecurity and financial concern during the COVID‐19 pandemic are associated with worse mental health. J Occup Environ Med. 2020;62(9):686‐691. 32890205 10.1097/JOM.0000000000001962

[42] Agterberg G , Passchier J . Stress among seamen. Psychol Rep. 1998;83:708‐710. 9819944 10.2466/pr0.1998.83.2.708

[43] Rhoades L , Eisenberger R . Perceived organizational support: a review of the literature. J Appl Psychol. 2002;87(4):698‐714. 12184574 10.1037/0021-9010.87.4.698

[44] Brun J‐P , Dugas N . An analysis of employee recognition: perspectives on human resources practices. Int J Hum Resour Manag. 2008;19(4):716‐730.

[45] Luthans K . Recognition: a powerful, but often overlooked, leadership tool to improve employee performance. J Leadersh Stud. 2000;7(1):31‐39.

[46] Madathil R , Heck NC , Schuldberg D . Burnout in psychiatric nursing: examining the interplay of autonomy, leadership style, and depressive symptoms. Arch Psychiatr Nurs. 2014;28(3):160‐166. 24856267 10.1016/j.apnu.2014.01.002

[47] Brooks SK , Greenberg N . Non‐deployment factors affecting psychological wellbeing in military personnel: literature review. J Ment Health. 2018;27(1):80‐90. 28132582 10.1080/09638237.2016.1276536

[48] Mikkelsen A , Saksvik PO , Landsbergis P . The impact of a participatory organizational intervention on job stress in community healthcare institutions. Work Stress. 2000;14:156‐170.

[49] Weston MJ . Strategies for enhancing autonomy and control over nursing practice. Online J Issues Nurs. 2010;15(1):10.

[50] Brooks SK , Greenberg N , Wessely S , Rubin GJ . Factors affecting healthcare workers' compliance with social and behavioural infection control measures during emerging infectious disease outbreaks: rapid evidence review. BMJ Open. 2021;11(8):e049857. 10.1136/bmjopen-2021-049857 PMC8370838 34400459

